# The Level of Agreement Among Medical Oncologists on Adjuvant Chemotherapy Decision for Breast Cancer in Pre and Post-Oncotype DX Settings

**DOI:** 10.7759/cureus.13298

**Published:** 2021-02-12

**Authors:** Abdulmohsen Alkushi, Ahmad Omair, Emad Masuadi, Ghaida Alamri, Atlal Abusanad, Nafisa Abdelhafiez, Amin E Mohamed, Omalkhair Abulkhair

**Affiliations:** 1 Pathology, King Abdulaziz Medical City of National Guard, Riyadh, SAU; 2 Pathology, College of Medicine, King Saud bin Abdulaziz University for Health Sciences & King Abdullah International Medical Research Center, Riyadh, SAU; 3 Pathology, College of Science & Health Professions, King Saud bin Abdulaziz University for Health Sciences & King Abdullah International Medical Research Center, Riyadh, SAU; 4 Research Unit/Biostatistics, College of Medicine, King Saud bin Abdulaziz University for Health Sciences & King Abdullah International Medical Research Center, Riyadh, SAU; 5 Medicine, College of Medicine, King Saud bin Abdulaziz University for Health Sciences & King Abdullah International Medical Research Center, Riyadh, SAU; 6 Medicine, King Abdulaziz University, Jeddah, SAU; 7 Medical Oncology, King Abdulaziz Medical City of National Guard, Riyadh, SAU; 8 Oncology, Suliman Alhabib Hospitals, Riyadh, SAU

**Keywords:** breast cancer, oncotype dx, adjuvant chemotherapy, recurrence risk, level of agreement

## Abstract

Introduction: The Oncotype DX assay plays an important role in the identification of the specific subset of hormone receptor (HR)-positive and node-negative breast cancer (BC) patients, who would benefit the most from adjuvant chemotherapy. The current study aimed at assessing the level of agreement among medical oncologists on adjuvant chemotherapy decisions before and after Oncotype DX, as well as the intra-observer agreement of each medical oncologist’s decision of prescribing adjuvant chemotherapy based on clinicopathological and immunohistochemical parameters only and followed by Oncotype DX recurrence score (RS) results.

Methods: A retrospective analysis of data related to clinicopathological and immunohistochemical parameters, and Oncotype DX RS result for 145 female, estrogen receptor (ER)-positive, HER2 negative, and both node-negative and positive BC patients was performed. Initially, the data without Oncotype DX RS was sent to 16 oncologists in multiple centers in the Middle East. After one week, the same data with the shuffling of cases were sent to the oncologists with the addition of the Oncotype DX RS result for each patient. The inter and intra-observer agreement (kappa and Fleiss multi-rater kappa) among oncologists' decision of prescribing adjuvant chemotherapy pre and post-Oncotype DX RS results were assessed. Oncotype DX risk scores were used as continuous variables as well as based on old RS grouping, categorized into low (0-17), intermediate (18-30), and high risk (≥ 31) groups. A test with a p-value of < 0 .05 will be considered statistically significant.

Results: The mean age ± SD of the cohort was 51.9 ± 9.4 years. Sixty-nine patients (47.6%) were premenopausal whereas 76 patients (52.4%) were postmenopausal. The mean Oncotype DX RS was 17.8 ± 8.6 and 54.5% had low recurrence risk (RR), 37.9% had intermediate RR and only 7.6% had high RR. The majority of our cases were grade two (53.1%) and T stage one (49%), whereas 29.7% had positive one to three lymph nodes. The addition of Oncotype DX results improved the agreement among oncologists' decision from fair to moderate (kappa = 0.52; p <0.001). On average, an oncologist’s decision of prescribing adjuvant chemotherapy pre and post-Oncotype DX had an agreement in 70.6% of the cases, with agreement observed mostly for cases where the initial decision of adjuvant chemotherapy was (no) and it was retained with post-Oncotype DX assay (46.1%), compared to 24.5% cases where the initial decision was (yes) and it was retained with post-Oncotype DX assay (kappa = 0.39; p <0.001). The addition of the Oncotype DX RS result avoided chemotherapy in 20.4% of cases and identified 9% of cases as candidates for adjuvant chemotherapy (kappa = 0.38; p <0.001). The disagreement was highest among cases with intermediate RR (33.6%) followed by high and low RR (31.3% and 21.6%) with a statistical significance of <0.001.

Conclusion: We conclude that the Oncotype DX RS significantly influenced the decision to prescribe adjuvant chemotherapy among HR-positive, HER2 negative, and both node-negative and positive patients, as it increased the level of agreement among oncologists and led to a decrease in the use of adjuvant chemotherapy compared to the pre-Oncotype recommendations.

## Introduction

Breast cancer (BC) is associated with a high incidence rate and is a major cause of mortality worldwide and in the Middle East [1]. Early diagnosis and adjuvant therapies have reduced the mortality rate despite high incidence [2]. Along with surgery, endocrine therapy (ET) and chemotherapy, or a combination of both, are the major systemic adjuvant therapies in the treatment of BC. Various studies have reported a significant role of adjuvant chemotherapy (AC) in decreasing the early recurrence of the disease [3]. It has been reported that out of the annually reported BC cases in the United States, the majority are hormone receptor (HR)-positive, less aggressive with less metastatic potential, and usually treated with ET [4]. Whereas HR-negative tumors are most likely treated with chemotherapy because of their aggressive nature and a greater tendency for metastasis [5]. A proportion of the HR-positive patients also requires AC to decrease their recurrence risk and improve survival as recommended by the National Surgical Adjuvant Breast and Bowel Project (NSABP)-20 trial, which found a 7% improvement in five-year recurrence-free survival by the addition of chemotherapy to ET [6].

Traditionally, the use of AC is based upon clinicopathological characteristics, including patient age, menopausal status, tumor hormonal status, human epidermal growth factor (HER2) status, Ki67 proliferation index, and tumor, nodes, and metastases (TNM) stage [7]. Identification of the specific subset of HR-positive and node-negative patients who would benefit the most from chemotherapy to reduce the risk of recurrence and improve survival is the main goal for oncologists in treating BC. This may reduce the unnecessary use of chemotherapy and its associated toxicity and morbidity. Similarly, it may limit under-treatment in patients who are likely to benefit from adding AC to endocrine therapy. Different multi-gene panels have been developed and applied in combination with other pathological variables to risk-stratify patients with early HR-positive, HER2-negative BC but Oncotype DX (ODx) is the only assay validated by various trials [8,9].

The ODx is a commercially available 21 gene assay (Genomic Health, Inc., Redwood City, CA) that uses a quantitative reverse transcription-polymerase chain reaction (RT-PCR) to determine long term recurrence risk (RR) for HR-positive and HER2-negative BC patients. It provides a continuous numeric recurrence score (RS) ranging from 0 to 100 or divided into low (0-17), intermediate (18-30), and high scores (≥ 31) [8]. The recent TAILORx data redefined these risk categories for < 50 years old patients into low risk (< 16), intermediate (16-25), and high-risk groups (> 25) [10]. The 21 genes assay includes 16 cancer genes subdivided into five proliferative genes (Ki67, STK15, Survivin, CCNB1, and MYBL2), five estrogen receptor (ER) genes (ER, PGR, BCL2, and SCUBE2), two HER2 genes (HER2 and GRB7), two invasive genes (MMP11 and CTSL2), and one gene from GTSM1; and remaining five reference genes including B-Actin, GAPDH, RPLPO, TFRC, and GUS genes. The formula for calculating the recurrence score from RT-PCR results gives the highest weight to the genes of proliferation, ER, and HER2 groups.

The ability of ODx to predict the recurrence risk has already been validated in the NSAB-B14 trial, which reported it to be an independent predictor even after adjusting for age and tumor size [8]. It can also independently predict mortality based on the risk categories [11]. Its use in identifying the patients with HR-positive and node-negative BC who would benefit from adding chemotherapy to ET based on RS has also been validated in the NSABP-20 trial [9].

The use of ODx is not limited to risk-stratify patients with node-negative BC, but it has been also applied in the management of node-positive HR-positive tumors. Although chemotherapy has been the standard of care for node-positive disease [12], several studies have reported that it may confer minimal survival benefits that are not outweighing the associated side effects [13]. Studies have shown that OncotypeDx can predict long term recurrence in both early node-negative and positive BC and identify the subset of node-positive BC patients who would benefit from the addition of AC to ET [14]. The significance of this assay in guiding AC can be appreciated by the fact that certain studies reported a change in the management plan in up to 44 cases; mostly led to omitting chemotherapy though it was initially planned [15].

The current study examined the level of agreement among medical oncologists on AC decisions before and after the ODx assay recurrence score results, and hence assessed the impact of the ODx recurrence score on the oncologist's decision to prescribe AC. This study also assessed the intra-observer agreement of each medical oncologist's decision of AC based upon clinicopathological and immunohistochemical parameters and post-ODx assay.

## Materials and methods

This is a cross-sectional study approved by the Institutional Review Board (IRB) involving a retrospective analysis of the pathology reports from 145 female BC patients by nonprobability consecutive sampling at King Abdulaziz Medical City (KAMC), Riyadh, and King Abdulaziz University Hospital, Jeddah. The data were obtained from the hospital electronic database, oncology database, and pathology reports and then reviewed and entered into the data collection sheet. All patients included were diagnosed with non-metastatic BC between 2012 and 2019 were positive for ER and negative for HER2, and had ODx recurrence score. BC patients with negative HR, positive HER2 status, metastatic disease, or no ODx assay were excluded from the study. 

The tumor sections for all 145 patients were sent to Genomic Health, Inc. for ODx multigene testing. The recurrence scores predicting the recurrence risk were received given as continuous variables. Histologic assessment of the sections was also done by qualified pathologists. Different clinicopathological parameters were assessed and recorded for each case and included patient’s age, menopausal status (premenopausal or postmenopausal), cell type (lobular or ductal), mitotic index (MI), tumor grade (assessed by Nottingham criteria), the tumor pathological stage (T stage), nodal status (N stage), metastatic status (M stage), and overall stage. 

Immunohistochemistry was used to assess the expression of hormonal receptors ER, progesterone receptor (PR), and HER2, as well as Ki67 using monoclonal antibodies (ER {clone SP1}, PR {clone 1E2}, HER2 {clone 4B5}, and Ki67 {clone MIB-1}). The immunostaining for these receptors was interpreted as per American Society of Clinical Oncology/College of American Pathologists (ASCO/CAP) guidelines [16,17]. ER and PR were each considered as positive when ≥ 1.0% of tumor cells get nuclear staining and negative when < 1.0% of tumor cells or no nuclear staining is observed. Based on ASCO/CAP guidelines, immunostaining for HER2 was categorized into 0, 1+, 2+, and 3+. The staining is interpreted as negative when there is either no staining or incomplete faint membrane staining in ≤ 10% of tumor cells (0) or when there was incomplete faint membrane staining in > 10% of tumor cells (1+) and interpreted as positive when there was complete intense circumferential membrane staining in > 10% of invasive cancer cells (3+). When the membrane staining was incomplete weak/moderate in > 10% of tumor cells, or when ≤ 10% of tumor cells show complete, intense, and circumferential membrane staining, the result was interpreted as Equivocal (2+). The equivocal HER2 stained samples were analyzed by fluorescence in situ hybridization (FISH) and only included if they come out to be negative. Ki67 proliferation index was based on the percentage of positive nuclear staining of neoplastic cells counted at the edge and center of the tumor and given as a continuous variable.

The data related to clinicopathological and immunohistochemical parameters for all included BC patients without ODx RS were sent to 16 medical oncologists from different tertiary centers in Saudi Arabia and other Middle Eastern countries. The data were sent in an Excel sheet without patient identification. Each oncologist was asked to fill their decision on AC blindly based on available clinicopathological and immunohistochemical data provided. Then, in a week, the same data were sent to the same group of medical oncologists after adding the ODx RS for each patient. The cases were shuffled and randomly arranged on the second run. This time, the medical oncologist was asked to fill their chemotherapy decision based on clinicopathological, immunohistochemical parameters, and ODx RS results. 

The descriptive statistics were given as mean ± SD or median with quartiles for numerical variables and as frequencies and percentages for categorical variables. In the analysis, the ODx risk scores were used as continuous variables as well as based on old RS grouping, categorized into low (0-17), intermediate (18-30), and high risk (≥ 31) groups. Microsoft Excel and IBM SPSS Statistics for Windows, Version 22.0 (IBM Corp., Armonk, NY) was used for data entry and analysis. The inter and intra-observer concordance rates are calculated to measure the degree of agreement among medical oncologists prior and post ODx RS results. A kappa value was calculated to assess the agreement between pre and post-Oncotype chemotherapy decisions and the inter and intra-observer concordance. Fleiss multi-rater kappa was calculated for assessing agreement among all 16 raters together. A kappa value of 0 will be considered as no agreement, 0.01-0.20 as slight agreement, 0.21-0.40 as fair agreement, 0.41-0.60 as moderate agreement, 0.61-0.80 as substantial agreement, and 0.81-0.99 as almost perfect agreement [18]. A test with a p-value of < 0 .05 was considered statistically significant.

## Results

A total of 145 female patients with BC who had ODx testing were included in this study. Of these, 138 cases were invasive ductal carcinoma (95.2%) and only four were invasive lobular carcinoma (4.8%). The mean age ± SD of the study population was 51.9 ± 9.4 years. Sixty-nine patients (47.6%) were premenopausal whereas 76 patients (52.4%) were postmenopausal. The mean ODx recurrence score of our cohort was 17.8 ± 8.6. Categorical distribution of the RS revealed 79 cases (54.5%) had low-risk (0-17), 55 (37.9%) had intermediate-risk (18-30), and only 11 (7.6%) had high-risk (31-100). The progesterone receptor was positive in 139 cases (95.9%) and negative in only six cases (4.1%). The mean ± SD of the Ki67 proliferation index in our cases was 16 ± 15. The majority of our cases had tumor grade two (53.1%), followed by grade one (33.8%), and the least was grade three (13.1%). The tumor T stage was one for 71 cases (49%), followed by stage two in 66 cases (45.5%), and stage three in only eight cases (5.5%). Nearly, 102 patients (70.3%) were node-negative (N0) whereas 43 patients (29.7%) had positive one to three lymph nodes (N1). Most of our cases (N = 81; 55.9%) were found to have an overall stage II followed by overall stage I (N = 56; 38.6%) and overall stage III (N = 8; 5.5%). The clinicopathological and immunohistochemical characteristics of the study cohort are summarized in Table 1.

**Table 1:** The baseline clinicopathological and immunohistochemical characteristics of 145 breast cancer patients *Continuous variables **Quartiles PR: progesterone receptor

Characteristics	Count (N)	Percent (%) of total
Menopause		
Premenopausal	69	47.6
Postmenopausal	76	52.4
Cell type		
Ductal	138	95.2
Lobular	7	4.8
Grade		
1	49	33.8
2	77	53.1
3	19	13.1
PR		
Positive	139	95.9
Negative	6	4.1
T stage		
1	71	49.0
2	66	45.5
3 and 4	8	5.5
N stage		
0	102	70.3
1 (1–3 nodes)	43	29.7
Overall stage		
Stage I	56	38.6
Stage II	81	55.9
Stage III	8	5.5
Recurrence score		
Low risk (1–17)	79	54.5
Intermediate risk (18–30)	55	37.9
High risk (31–100)	11	7.6
	Mean ± SD	Median (Q1–Q3)**
Recurrence score*	17.8 ± 8.6	17 (12–23)
Age*	51.9 ± 9.4	51 (45–58)
Ki67*	16.0 ± 15.0	10 (5–22.5)

The inter-observer agreement among 16 oncologists regarding the decision to give chemotherapy was found to be fair (kappa = 0.38) when the decision was based on clinicopathological and immunohistochemical data only. On the addition of ODx RS to the previously provided information, the agreement improved to moderate (kappa = 0.52). The agreement was found to be statistically significant with a p-value of <0.001 for both pre and post-ODx chemotherapy decisions (Table 2).

**Table 2:** Inter-observer agreement among 16 oncologists, pre and post-Oncotype DX assay *Fleiss multi-rater kappa for 16 raters CI: confidence interval

Overall agreement	Kappa*	P-value	95% CI of kappa	
			Lower	Upper
Pre-Oncotype DX assay	0.38	<0.001	0.38	0.39
Post-Oncotype DX assay	0.52	<0.001	0.52	0.52

We also analyzed the intra-observer agreement for each of the 16 oncologist’s decisions of prescribing AC before and after knowing the ODx RS information. Table 3 describes our findings in detail. On average, an oncologist had an agreement in 70.6% of the cases when comparing the decision of prescribing AC pre and post ODx result with an average kappa value of 0.38 indicating a fair agreement. The percentage of cases where each rater achieved an agreement in pre and post-ODx chemotherapy decision ranged from 48.3% to 91.1% with the least agreement found with kappa = 0.09 (slight agreement) and the strongest agreement was found with kappa 0.77 (substantial agreement). The most agreement was observed for cases where the initial decision of AC was (no) and it was retained with the post-ODx assay (46.1%), followed by 24.5% cases where the initial decision of prescribing AC was (yes), and it was retained with the post-ODx assay. The observed agreement was statistically significant in 15 out of 16 oncologists (p = 0.03-p < 0.001); but for one oncologist, it was statistically not significant (p = 0.11). This oncologist has the least agreement (kappa = 0.09) among only 48.3% of cases. On average, an oncologist changed the decision in 20.4% of cases from yes to no for chemotherapy (range: 0-46.2) and in 9% of cases from no to yes (range: 4.1-22.1). 

**Table 3:** Intra-observer agreement of 16 individual raters between pre and post-Oncotype DX decision for the use of adjuvant chemotherapy

	Disagreement (%)		Agreement (%)		Total agreement (%)	Kappa	95% CI of kappa		
Raters	Yes-No	No-Yes	No-No	Yes-Yes	No-No and Yes-Yes		Lower	Upper	P-value
Rater 1	13.1	13.8	65.5	7.6	73.1	0.19	0.01	0.37	0.02
Rater 2	36.6	4.1	33.8	25.5	59.3	0.26	0.14	0.38	< 0.001
Rater 3	18.6	6.2	53.8	21.4	75.2	0.46	0.31	0.60	< 0.001
Rater 4	14.5	13.1	51.0	21.4	72.4	0.40	0.24	0.55	< 0.001
Rater 5	17.2	12.4	42.8	27.6	70.4	0.39	0.24	0.54	< 0.001
Rater 6	12.4	8.3	64.8	14.5	79.3	0.45	0.28	0.61	< 0.001
Rater 7	19.3	4.8	39.3	36.6	75.9	0.53	0.40	0.66	< 0.001
Rater 8	24.8	7.6	51.0	16.6	67.6	0.29	0.14	0.44	< 0.001
Rater 9	30.3	4.1	33.8	31.7	65.5	0.35	0.23	0.48	< 0.001
Rater 10	6.2	6.2	74.5	13.1	87.6	0.60	0.44	0.77	< 0.001
Rater 11	6.9	22.1	51.0	20.0	71.0	0.38	0.23	0.52	< 0.001
Rater 12	0.0	9.0	21.4	69.7	91.1	0.77	0.65	0.89	< 0.001
Rater 13	35.2	9.7	27.6	27.6	55.2	0.16	0.02	0.30	0.03
Rater 14	46.2	5.5	32.4	15.9	48.3	0.09	0.00	0.20	0.11
Rater 15	29.7	6.9	41.4	22.1	63.5	0.28	0.14	0.42	< 0.001
Rater 16	15.2	10.3	53.8	20.7	74.5	0.43	0.27	0.58	< 0.001
Average	20.4	9.0	46.1	24.5	70.6	0.38	0.01	0.37	0.01

Table 4 also describes the same findings described above for all when 16 oncologists' decisions were grouped. The agreement has tested the pre and post-ODx assay 2320 times (16 oncologists assessing 145 patients) and revealed fair agreement (kappa = 0.39) in 70.6% cases with a statistically significant p-value of < 0.001. 

**Table 4:** Agreement and disagreement between pre and post-Oncotype DX RS chemotherapy decision RS: recurrence score No. of valid tests: 145x16 = 2320; p-value < 0.001; kappa = 0.39; 95% CI = (0.35–0.43)

Decision pre-Oncotype DX RS		Decision post-Oncotype DX RS		Total
		No	Yes	
No	N	1070	209	1279
	% of total	46.1	9.0	55.1
Yes	N	473	568	1041
	% of total	20.4	24.5	44.9
Total	N	1543	777	2320
	% of total	66.5	33.5	100

We also grouped and analyzed the agreement and disagreement on chemotherapy decision that was made by all oncologists pre and post-ODx assay, where the RS was categorized into low, intermediate, and high-risk categories (Table 5). For the cases in the low-risk category, the agreement was observed in 73.4% of cases, and disagreement was observed in only 26.6%. Contrary to this, among cases with intermediate-risk of recurrence, the agreement was observed in 66.4% and disagreement was observed in only 33.6% of the cases. Among the cases with high-risk RS, the agreement was observed in 68.7% of cases, and disagreement was observed in only 31.3%. 

**Table 5:** Agreement and disagreement between pre and post-Oncotype decision when RS is categorized RS: recurrence score No. of valid tests: 145x16 = 2320; p < 0.001

Recurrence Score	Decision pre-Oncotype DX RS		Decision post-Oncotype DX RS		Total
			No	Yes	
Low-risk (1-17)	No	Count	781	19	800
		% of total	63.4	1.5	64.9
	Yes	Count	309	123	432
		% of total	25.1	10	35.1
	Total	Count	1090	142	1232
		% of total	88.5	11.5	100
Intermediate-risk (18-30)	No	Count	252	135	387
		% of total	28.6	15.3	44.0
	Yes	Count	160	333	493
		% of total	18.2	37.8	56.0
	Total	Count	412	468	880
		% of total	46.8	53.2	100
High-risk (31-100)	No	Count	10	55	65
		% of total	5.7	31.3	36.9
	Yes	Count	0	111	111
		% of total	0	63.1	63.1
	Total	Count	10	166	176
		% of total	5.7	94.3	100

## Discussion

Several international cancer societies and institutes recommend the use of ODx assay to risk-stratify patients with early HR-positive and HER2-negative BC and to predict the potential benefit of adding AC to ET, thus avoiding the use of expensive and toxic chemotherapy unnecessarily while ensuring the maximum benefit in patients who are at high risk of recurrence [12]. The role of ODx assay in predicting additional benefit from chemotherapy in node-positive cases has also been established [14]. This study is the first of its kind that assessed the impact of ODx recurrence score on the oncologists’ decision of prescribing AC individually and as a group. It examined the level of agreement in decision among 16 medical oncologists and the intra-observer agreement of each medical oncologist’s decision pre and post-ODx assay. We report a significant impact of ODx RS result on the oncologist’s decision-making in prescribing AC. The test led to an improvement in the interobserver level of agreement and prevented the use of AC in 20.4% of the cases. This study adds to the scarce data on the impact of ODx on the clinical practice of BC treatment in the Middle East.

The frequency of our patients in low, intermediate, and high-risk groups (54.5%, 37.9%, and 7.6%) is similar to what has been reported in a prior Middle Eastern cohort (53.2%, 40.4%, and 6.4%) [1]. Another study from the same region reported a relatively lower frequency for the cases in the intermediate-risk group (27.8%) [19]. A meta-analysis of 21 international studies reported average frequencies of patients in low, intermediate, and high-risk groups to be 48.8%, 39%, and 12.2%, respectively [20]. In comparison, our study revealed a comparatively higher percentage of those in the low-risk category and a lower percentage of patients in the high-risk group, and this may be due to our institute’s filtering process which aids in reducing the ordering of the expensive assay for patients who would not benefit from it. These studies followed the traditional classification for stratification of RS scores into the three risk categories (low: < 18, intermediate: 18-30, and high: ≥ 31). Although a new classification has been proposed by the TAILORx data (low: < 11, intermediate: 11-25, and high: > 25) [10]. Our study applied the traditional classification which is supported by the observation of Zekri et al. that found no statistically significant difference in prescribing AC by three blinded oncologists using the two classifications [21]. Controversy and challenge in the management of patients with intermediate RS of 11 to 17 exist when applying the updated classification. Nonetheless, studies have reported that RS can guide the AC decision even in the intermediate-risk group when used as a continuous variable [22]. Therefore, we used the ODx RS both as a continuous variable and classified traditionally into three risk categories to explore the impact of the assay on clinical decision making through assessing the level of agreement among oncologists.

This study reports a statistically significant increase in the level of agreement among 16 oncologists on the use of AC when they were provided with the ODx RS compared to pre-Oncotype assay. The level of agreement was fair based on the clinicopathological and immunohistochemical data but improved to a moderate agreement on the addition of the RS information. Reliance on the clinicopathological and immunohistochemical data alone can lead to an underestimation of survival and hence encourages the use of chemotherapy [23]. 

Using the RS as a continuous variable, we report that on average each oncologist had an intra-agreement pre and post-ODx assay in two-thirds of the cases, mostly (46.1%) for cases with a pre-ODx decision of omitting AC (i.e., No to No agreement). On the other hand, on average, in 20.4% of cases, the assay modified the oncologist’s recommendation for chemotherapy from yes to no. This highlights the importance of the assay in limiting the unnecessary use of chemotherapy and its potential side effects that might be associated with poor life quality among BC patients. We also reported that on average in 9% of the cases the ODx score predicted the patients who are candidates for AC, where the oncologist’s pre-ODx decision was to forgo chemotherapy. This further reinforces the significance of this assay in decreasing mortality among early-stage BC patients who would benefit from AC but would have not been treated properly in the absence of the ODx RS. Studies have reported that the ODx RS does not only predict the RR but can also predict mortality independently of other covariates [11]. In line with our findings, a recent study also reported an impact of the assay on AC decision, leading to a change in the decision of omitting chemotherapy in 37% of the cases where chemotherapy was recommended pre-ODx, and in 14.8% of the cases where chemotherapy was prescribed after the decision was changed to yes to chemotherapy from an initial decision of a no-recommendation [24]. Compared to our study they reported a higher percentage of patients with a change in the decision for both groups. This could mainly be because in their study the change is based on a decision from a single medical oncologist in a multidisciplinary team, whereas we report an average percentage of cases with a change in the decision based upon decisions from 16 oncologists in multiple centers in the Middle East. 

Observing the impact of ODx on the individual oncologist decision; one oncologist changed the decision of prescribing chemotherapy in 46.2% of the cases. This was the highest percentage of decision difference among the oncologists' cohort. While ODx guided another oncologist to change the decision from no to yes in 22.2% of the patients. This observation of a net reduction in the recommendations for the use of chemotherapy post-ODx assay has been reported by many studies including Jaafar et al. who reported a 50% reduction in chemotherapy recommendation post-ODx in 47 node-negative Middle Eastern patients with BC [1]. 

Although tumor size, grade, stage, and nodal status are considered independent risk factors, it could be the biological makeup of the tumor that may largely lead to recurrence and metastasis with a smaller size, low grade, and negative nodal involvement. These patients could benefit from chemotherapy and the ODx assay RS can guide the oncologist to change the pre-Oncotype decision of only endocrine therapy to chemo-endocrine treatment. Such patients in our cohort accounted for 9% of cases where oncologist changed the recommendation in favor of chemotherapy post-ODx. We observed that the oncologists switched their decision on prescribing AC from yes to no in a higher percentage of the cases than switching their decision from no to yes after the reveal of the ODx result. In other words, the change to a decision of chemotherapy administration was less frequent than the change to a decision of omitting chemotherapy. This is consistent with those reported in the region as well as internationally [1,24,25]. Rizki et al. also reported that the precision of the ODx score in guiding the decision to use AC is much superior to other clinicopathological tools [26]. Studies have reported that ODx is being used more frequently now compared to when it was initially made available and this has shifted the trends in using AC among oncologists. A study from Ireland reported a decrease in the use of chemotherapy during the periods where Oncotype assay was used and even during the period when it was not available for some time [27]. Even though ODx is a costly assay, studies have reported it to be more cost-effective by its impact on the decrease in the use of AC [28].

We report a better agreement of oncologist’s AC decisions among low-risk and high-risk groups (73.4% and 68.7%) as compared to the intermediate-risk group (66.4%). On average, an oncologist changed the pre-ODx decision of prescribing chemotherapy to the decision of omitting it post-ODx in 25.1% of the low-risk category, 18.2% of the intermediate-risk category, and none among the high-risk category. This is in line with the reported trends of the intermediate and high-risk group to receive chemotherapy more frequently than the low-risk group [19]. Along with increasing the confidence of oncologists in prescribing chemotherapy, ODx also increases the satisfaction of the patient with the therapeutic decision. Anxiety among patients is common upon knowing that they may need AC and having an evidence-based tool to assist in the decision-making provides an assurance and promotes compliance [25]. 

Evolving evidence supports the use of ODx in early node-positive BC that is HR-positive and HER2 negative. Bello et al. reported a similar distribution of RS scores among node-negative and positive patients and highlighted the fact that a subset of those patients with nodal involvement may not benefit from AC that is usually decided pre ODx, based on nodal status only [29]. Interestingly, one-third of the patients in our study had one to three lymph nodes involved. Hanna et al. reported that most of their node-positive cases had low recurrence risk (RS < 18) [30]. Our findings support their observation, as the mean RS of our node-positive cases was 17.3 ± 8 compared to the mean RS of 18 ± 9 for node-negative patients and hence reaffirms the role of ODx assay in node-positive cases who would not benefit from AC. 

The strengths of this study are a relatively larger sample size compared to previous studies from the region, being the first study of this type to assess the level of interobserver and intra-observer agreement among 16 highly qualified medical oncologists in multiple centers in the Middle East and the inclusion of node-positive cases as well. Limitations of this study include its retrospective design; lack of long-term follow-up data and information about the patient outcome.

## Conclusions

We conclude that ODx RS significantly influenced the decision to prescribe AC among HR-positive, HER2-negative, and both node-negative and positive patients. It substantially increased the level of agreement among the cohort oncologists and led to a decrease in the use of AC compared to the pre-Oncotype recommendations. Similarly, it led to the identification of cases that would benefit most and are candidates for adjuvant chemotherapy.

This study adds to the data on the impact of ODx on clinical practice and further conclude that ODx is a useful additional guiding tool for oncologists for prescribing adjuvant chemotherapy in breast cancer patients who met the selection criteria.
